# Comparing brown mite, *Bryobia rubrioculus* Scheuten (Acari: Tetranychidae) sampling methods in almond orchards in California

**DOI:** 10.1007/s10493-026-01119-w

**Published:** 2026-02-28

**Authors:** Buddhi B. Achhami, Jhalendra P. Rijal

**Affiliations:** 1 California Department of Food and Agriculture, Sacramento, CA USA; 2 University of California, Agriculture and Natural Resources & Statewide IPM Program, Davis, CA USA

**Keywords:** Tree-band, Shoot tuft, Bryobia rubrioculus, Leaf sampling

## Abstract

Several arthropod pests infest almond orchards in California’s Central Valley, United States, which produces ~ 80% of the almonds globally. Some are key pests, while others are minor but can cause economic damage due to fluctuations in environmental factors from year to year. Thus, keeping pest levels below the economic injury level is key for sustainable and profitable almond production. As a part of the integrated pest management (IPM) concept, record-keeping for the occurrence of various pests using an adequate sampling plan aids in developing a future pest management program. Thus, we tested sampling methods for the brown mite, *Bryobia rubrioculus* Scheuten, which is becoming an increasing problem in almond orchards in California. We sampled three almond orchards in the northern San Joaquin Valley from February to July at bi-weekly intervals. We used a tree-band trap on the tree trunk to collect mites from the lower part of the canopy and trunk, and leaf and shoot tuft samplings to collect mites from the top canopy of the plants. Consistent with all orchards, we collected the highest number of mites from shoot tufts than from tree bands, and the lowest number from leaves. Our results provide a foundation for monitoring *B. rubrioculus* populations in commercial almond orchards in California.

## Introduction

Almond, *Prunus dulcis* (Miller) D.A. Webb, stands as the world’s foremost tree nut crop, surpassing a total production of 1.36 million metric tons globally (INC 2021). While almond cultivation is adaptable to diverse regions, the primary commercial production is confined to areas featuring Mediterranean climates. These climates are characterized by mild winters and hot, dry summers. Key production zones include Mediterranean countries such as Italy, Spain, Morocco, Turkey, and Portugal, Middle Eastern countries like Iran and Turkey, and select nations in the Southern Hemisphere — Chile, Argentina, South Africa, and Australia, and in North America - the Central Valley of the state of California of the United States (Gradziel et al. 2017). California contributes over 79% of global production (INC 2021). The U.S. maintains approximately 660,000 hectares of almond orchards, primarily concentrated in California’s Central Valley (NASS 2023), with the farm gate value of the nut exceeding $5 billion USD (CDFA 2023).

Over two dozen insect and arthropod species are considered pests in almond orchards in California (Haviland et al. 2017). Several species of mite (Class: Arachnidae) feed on the leaves and have been reported as pests of almonds in the Central Valley of California (Andrews and Barnes 1981; Haviland et al. 2017). Those include spider mites (Acari: Tetranychidae) such as Pacific spider mite (*Tetranychus pacificus* McGregor), two-spotted spider mite (*T. urticae* Koch), and strawberry spider mite (*T. turkestani* Ugarov and Nikolskii), European red mites, *Panonychus ulmi* (Koch), citrus red mite, *Panonychus citri* (McGregor), and eriophyid mites (Acari: Eriophyidae) such as peach silver mite (*Aculus cornutus* Banks), and brown mite, *Bryobia rubrioculus* Scheuten (Acari: Tetranychidae). Among them, *T. pacificus* and *T. urticae* are key pests and cause severe leaf damage and leaf drop (Barnes and Curtis 1979; Wilson et al. 1984; Strand 2002; Haviland et al. 2017), which can impact the crop yield in the following year (Barnes and Andrews 1978). However, *B. rubrioculus* is becoming an economically important pest of almonds in the Central Valley and warrants miticide applications in many instances (JPR, Personal Observation). *Bryobia rubrioculus* is a pest of almonds and many other deciduous fruit trees in the United States (Summers 1950; Pritchard and Baker 1952) and other almond-producing countries in Asia and Europe (Osakabe et al. 2000; Keshavarz Jamshidian et al. 205; González-Zamora 0^2022^).

*Bryobia rubrioculus* is the largest mite that feeds on almond leaves. Adults are dull reddish brown with dark orange markings. The body is flat with long legs. Like other pest mites, *B. rubrioculus* inserts its stylet into the upper surface of the leaves and damages mesophyll palisade cells just beneath the upper epidermis and consumes the content of mesophyll cells (Pane and Lee 2002). The feeding on the leaves results in a mottled appearance in the beginning and ultimately results in chlorotic patches on the leaf due to dead or air-filled cells (Summers and Stocking 1972). Almond plants can recover mite-damaged cells of the leaves under low infestation density (Summers and Stocking 1972); however, a heavy infestation can remove a significant amount of chlorophyll content from leaves and likely impair the photosynthesis process, as well as trigger leaf drops and eventually impact crop yield (Haviland et al. 2017).

Like many arthropod pests, environmental factors, especially temperature, play a significant role in the abundance of *B. rubrioculus* in almonds and crop damage by them. Environmental temperatures between 21.1 °C and 29.4 °C (= 70–85^o^F) are favorable for *B. rubrioculus* activity and feeding. However, they avoid unshaded leaves, especially when the temperature reaches the upper range (Summers and Baker 1952; Summers and Stocking 1972). Depending on the years and weather conditions, *B. rubrioculus* can have up to three generations per year in California (Summers 1950; Haviland et al. 2017) and other parts of the world (Keshavarz Jamshidian et al. 2005). As temperature increases beyond the upper thresholds, feeding activities cease, and summer eggs go into diapause (Summers 1950).

Seasonal activity of *B. rubrioculus* can begin as soon as the almond tree blooms when nymphs hatch from the overwintering eggs. As the summer progresses, the elevated temperature causes feeding activities by nymphs and adults to cease and forces the eggs into diapause (Summers 1950). However, high population density for a relatively short period can deplete chlorophyll in new flushes, causing severe damage to growth and development. Thus, early detection and population estimation are key to managing this pest in almond orchards.

There have not been any recent publications or guidance on sampling methods for *B. rubrioculus* in almonds, as all studies have been focused on web-spinning spider mites, *Tetranychus* spp. In the early 1950 s, there were attempts to develop sampling methods for brown mites using twig samples (Summers and Bakers 1952), visually counting (Wilson et al. 1984), beating on twigs and branches, and collecting from the trunk (Pritchard and Baker 1952). However, these studies did not compare the twig sampling method with leaf and other sampling methods. Also, there have been vast differences in varieties of almonds, production systems, and environmental conditions. In the early 1990 s, there were reports of using tree-band sampling methods to reduce *T. pacificus* populations by excluding the overwintering female from moving up the tree from the ground (Zalom 1994). For this, a layer of duct tape (5 cm wide) was wrapped on a tree trunk, and tanglefoot was applied on top to trap moving mites. Since the trap was designed for *T. pacificus* movement, in our study, we modified the trap and used the inner sticky surface of the tape to capture mobile *B. rubrioculus* on the tree trunk – tree-band sampling. In addition, we compared the twig sampling method (hereafter called shoot tuft sampling) with the other two methods – leaf sampling and tree-band sampling. The implications of sampling methods to monitor brown mite populations during the season in almond orchards are discussed.

## Materials and methods

### Sampling location

Mite samplings were conducted from three orchard locations (Modesto, 37°34’50.2"N 120°59’29.1"W; Oakdale, 37°50’02.3"N 120°50’24.3"W; Turlock, 37°31’54.8"N 120°49’20.4"W), representing the northern San Joaquin Valley region, California. These orchards were managed conventionally according to standard practices, including planting, irrigation, cultural management, and pest management, as described in the almond production manual (Micke 1996).

In Modesto, the sampling was conducted from the last week of January 2018 (Week 5) through June (Week 26), while in Turlock and Oakdale, the sampling started from the first week of March (Week 10) through the last week of June (Week 26).

### Tree-band sampling

We used a layer of 2-inch (= 5.08 cm) wide cloth duct tape (Scotch™, 3 M Company, Saint Paul, MN) to create a band by encircling the tree trunk at 45–60 cm high from the soil surface and below the tree crotch, depending on the height of the tree. The sticky surface of the band was attached to the bark but still allowed the mites to move up and down and, during the process, get caught in the sticky surface. The tree bands were collected and replaced every two weeks, and the number of brown mite adults and nymphs was counted. Eight randomly selected almond trees from the orchard edge were used for each location to apply the tree band.

### Shoot tuft sampling

Since *B. rubrioculus* activities, especially during daylight hours, are not necessarily on the leaves, shoot tuft sampling was used. The tuft is the terminal portion (~ 15 cm) of the almond growing shoot, consisting of bunches of leaves, spurs, and buds. For sampling, the tuft of the individual shoot top was jarred three times onto letter-size (22 × 28 cm) white copy paper to dislodge the *B. rubrioculus* inhabiting the tuft. The number of mites on the white copy paper was counted. In order to address potential variations in mite abundance, ten trees representing the edge (border row trees, *n* = 5) and interior (i.e., 10 rows inside the orchard, equivalent to 60 m inside from the border; *n* = 5) of the orchard were selected for the shoot tuft sampling in all sites. Five tufts from each of the selected ten trees per site were sampled for *B. rubrioculus* on each sampling date.

### Leaf sampling

Since there are no leaf sampling guidelines developed for *B. rubrioculus*, leaf sampling to detect *B. rubrioculus* activities was conducted following the University of California Integrated Pest Management (UCIPM) Guidelines for web spinning spider mites (Haviland et al. 2017). From all three orchard sites, the same five trees from the border and five trees from the interior were used. Fifteen leaves from each of those two groups of five trees were taken. Then, the leaves were visually checked using a 10x hand lens, and the number of *B. rubrioculus* present in the leaves was recorded.

### Data analysis

First, we aggregated total adult mites caught by week and by sampling methods (leaf, shoot tuft, and tree-band) and orchard locations (Oakdale, Turlock, and Modesto). We first used Generalized Linear Models (GLM) with zero-inflated Poisson error (Long 1997) to predict the number of adults caught at different sampling weeks and locations. Next, we averaged the total adults by sampling week and compared the mean number of adults by sampling sites (leaf, tree band, and shoot tuft) in individual orchard locations. We used a generalized linear model (GLM) with quasipoisson distribution. The model included orchard locations and sampling sites as predictors with interaction terms between these variables. We also assessed the significance of the model parameters using a Type III analysis of variance (ANOVA) using the Car package. Additionally, to examine the model’s fit, we performed the test using the hnp package from DHARMa (Hartig 2024). We used emmeans package (Lenth 2024) for mean separation. All data analysis and graphs were prepared in R version 4.0.5 (Everitt and Hothorn 2010; R Development Core Team 2021).

## Results

**Fig. 1:**
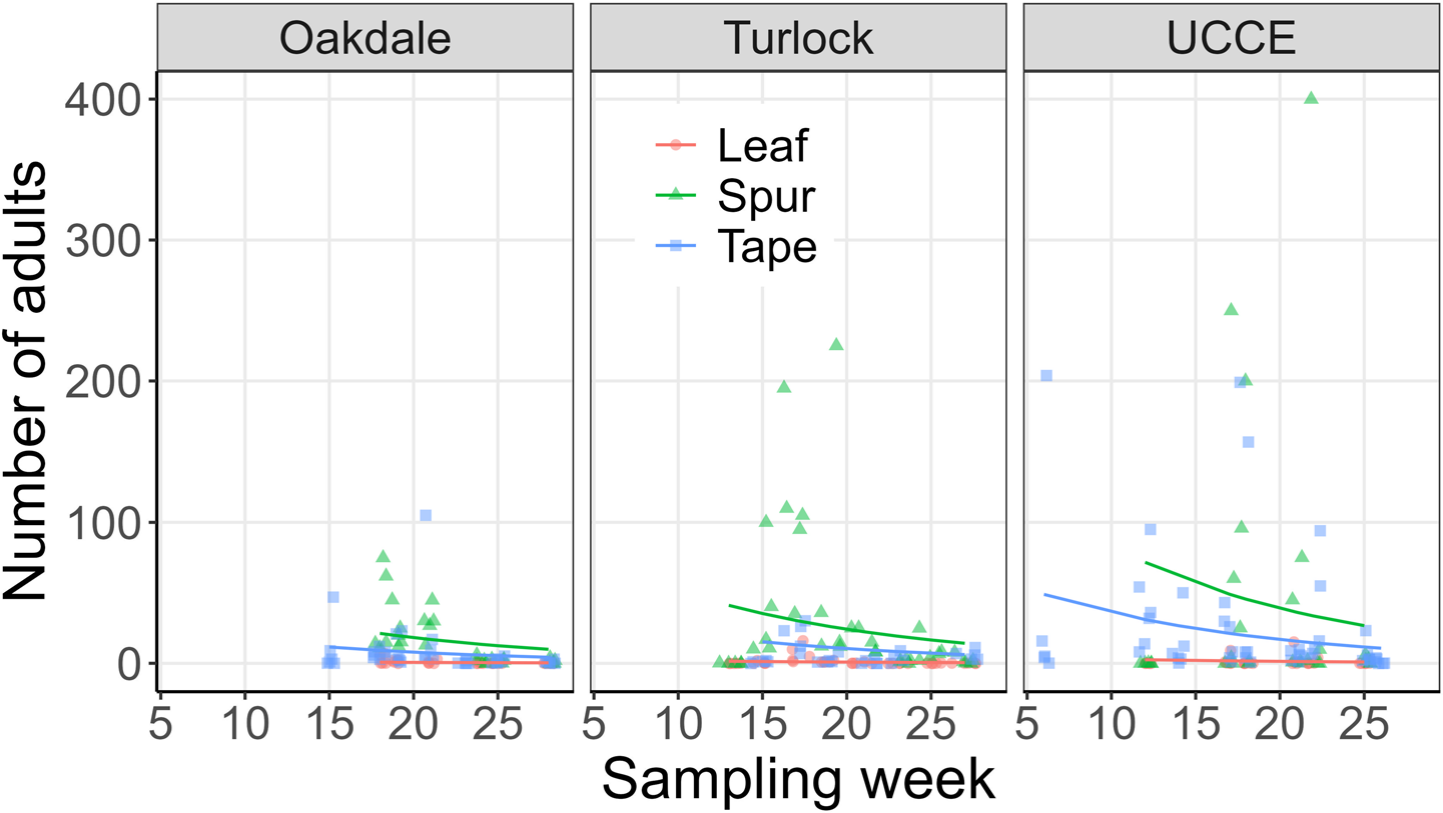
Seasonal trend of adult *B. rubrioculus* captured using three sampling methods across three almond orchard locations. Sampling week 5 represents the week from January 29 to February 4, 2018

Seasonal trend of *B. rubrioculus* capture. A total of 4,693 adults were counted (orchard location: Oakdale – 809; Turlock – 1,375; and Modesto – 2,510). The trend of catching adults was the lowest in leaf compared to shoot tuft and tree-band samplings across all orchard locations (Fig. 1). The trend line indicated that the overall mite density was higher in the earlier portion (Week 5–10) of the sampling season than in the later portion (i.e., week 25–30), regardless of the sampling methods (Fig. 1).

**Fig. 2:**
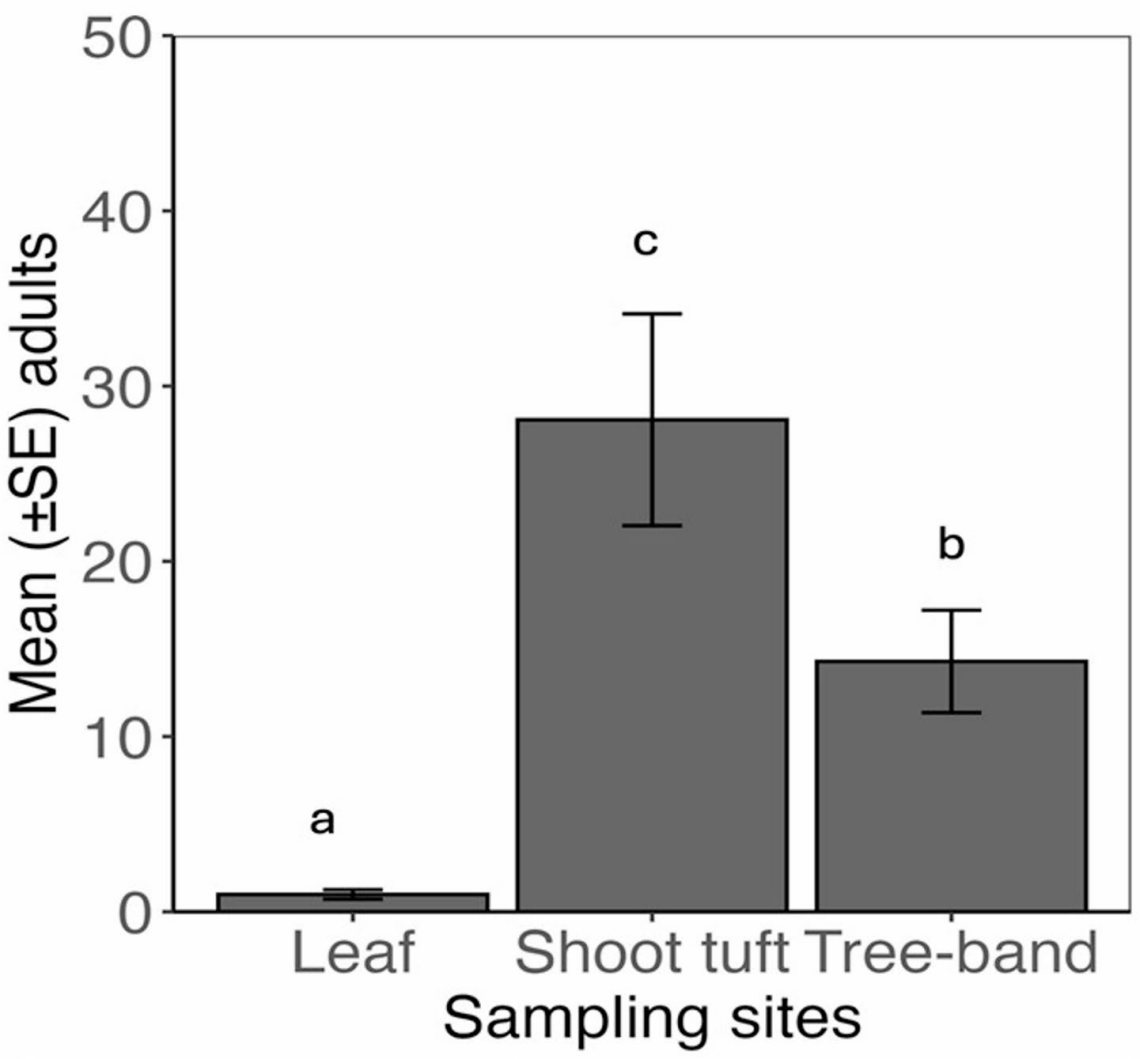
Mean seasonal total number of adult *B. rubrioculus* comparison by sampling site (leaf, shoot tuft, tree-band) across orchard location. Means not sharing the same letter are significantly different within each sampling site based on posthoc comparisons (*p* = 0.05)

Abundance of *B. rubrioculus*. The mean number of *B. rubrioculus* counted on the shoot tuft was 28, followed by tree-band 14, and on leaves < 1. By orchard location, the mean number of adult mites captured was not significant (LR Chisq = 0.03, df = 2, *p* = 0.98), sampling sites on tree was significant (LR Chisq = 16.47, df = 2, *p* = 0.002), and interactions between orchard location and sampling sites were not significantly different (LR Chisq = 1.68, df = 4, *p* = 0.79). Leaf (0.99 + 0.2 mean + SE), shoot tuft (28 + 6.4, mean + SE), and tree band (14.28 + 2.9, mean + SE) (Fig. 2).

## Discussion

*Bryobia rubrioculus* has been an increasing problem in almond orchards in California, and there is a need for developing a reliable monitoring tool to fit into the Integrated Pest Management (IPM) program. Currently available monitoring tool is limited to recording the presence-absence of overwintering *B. rubrioculus* mite eggs following the dormant spur sampling method (Haviland et al. 2017). However, there is a lack of sampling methods and guidelines for the growing season based on the seasonal mite population. Our study addressed this gap, studied the seasonal dynamics using multiple sampling methods, and recommended that the shoot turf sampling is the best method to assess the *B. rubrioculus* population during the growing season. *B. rubrioculus* overwinters as eggs on twigs, most often at the junction of two previous seasons’ wood (Haviland et al. 2017). Eggs hatch around bloom time, after which nymphs disperse to leaves and twigs to feed (Summers and Baker 1952). Feeding activity and damage are most significant from spring through early summer in the northern Central Valley of California (Summers and Baker 1952).

The early-season population of *Bryobia rubrioculus* originates from twigs, as this species overwinters as eggs deposited in bark crevices, at the base of buds and spurs. Egg hatch is closely synchronized with leaf and flower bud break, making tender developing tissues the preferred initial feeding sites in spring (Gratwick 1992). Our results reflected a similar trend, with mite abundance peaking from February to May (weeks 10–20) and declining by June–July (weeks 22–30). Movement intensity of *B. rubrioculus* within trees for food or shelter can vary across localities but occurs primarily between leaves and twigs (Summers 1950; Anderson and Morgan 1958).

In contrast to web-spinning spider mite pests such as *Tetranychus urticae* Koch and *T. pacificus* McGregor, which thrive under hot and dry conditions, *B. rubrioculus* populations in almond and other tree hosts tend to reach damaging levels in years with moist winters and relatively cool springs. The seasonal trends observed in our study showed that adult abundance peaked early in the season (March–May) and declined steadily during the summer months (June–July). This pattern reflects the typical biology of *B. rubrioculus*, which exhibits higher activity and abundance in spring during shoot and leaf expansion, followed by population reductions in summer as hot and dry conditions prevail. Similar early-season population peaks for *Bryobia* mites have been reported in other studies due to favorable environmental and weather conditions (Gratwick 1992).

**Fig. 3:**
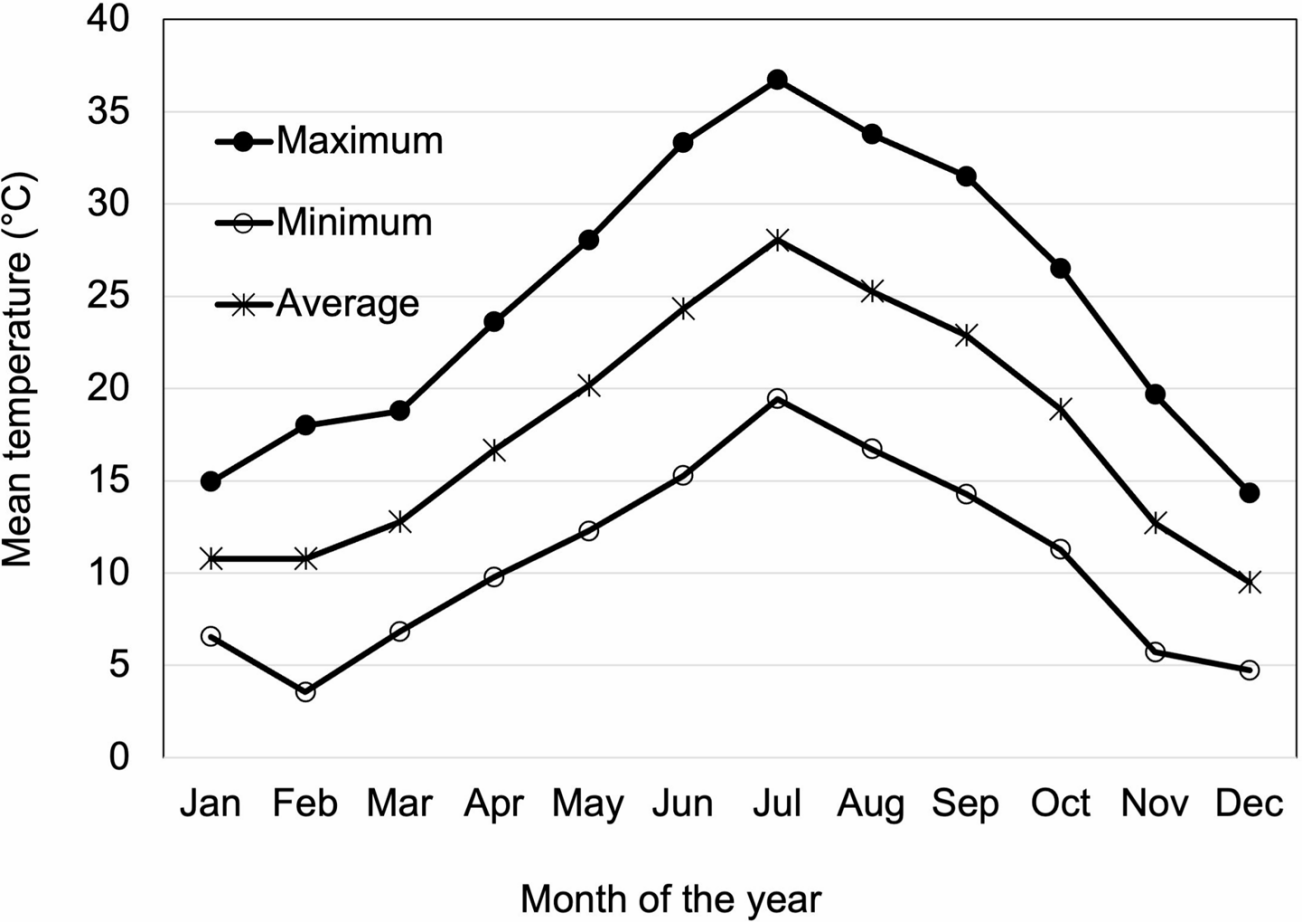
Average monthly maximum temperatures of Modesto, CA, representing the three almond orchard se weather. The data was retrieved from the National Weather Service website, ithttps://www.weather.gov/wrh/climate

Weather data for Stanislaus County (Modesto, CA), where all orchard sites were located, match the seasonal dynamics reported in this study (Fig. 3). These data show that June through August are the warmest months of the year, coinciding with minimal *B. rubrioculus* activity in our study. Notably, the mean temperature remained below 20 °C through May but increased to approximately 25 °C in June, supporting the observed seasonal decline in mite abundance (Fig. 3).

Our study is the first report of using tree trunk bands in detecting the *B. rubrioculus* population in almond orchards in California. While earlier studies reported movement of *B. rubrioculus* between leaves and twigs, none mentioned vertical movement along tree trunks. This could be due to the discrepancy in using the scientific name of brown mites. Pritchard and Baker (1952), Summers and Baker (1952), and Anderson and Morgan (1958) referred to the brown mite as *Bryobia praetiosa* in some literature, while still referring to it as the pest of almonds and other deciduous fruit trees. Historically, *B. praetiosa* was also referred to as “brown mite,” “almond brown mite,” and “clover mite,” reflecting confusion over host range and taxonomy. Given the infestation and prevalence of this pest in the almond orchard, we believe that in our study, the tree-band sampling method mostly captured *B. rubrioculus.* However, we also think the tree-band sampling method may not be the most reliable method for monitoring seasonal *B. rubrioculus* population due to logistical challenges in applying the bands on the tree, low trapping efficiency, and the potential of capturing multiple species of mites and other arthropods that may not be active pests, or economically important pests, but present in tree trunks.

In the comparison study among sampling methods, consistently higher numbers of *B rubrioculus* mites were detected on the shoot tuft compared to the tree band or leaf sampling methods. This trend suggests that shoot tufts might provide a more suitable microhabitat and tender leaves and tissues as favorable food resources for *B. rubrioculus* survival. The leaf sampling method yielded very low mite counts (< 1 mites/leaf) – 28 times lower than the tuft sampling, highlighting the limited reliability of leaf sampling for detecting *B. rubrioculus* adults in the field. Other studies of developing detection methods for *B. rubrioculus* for other tree crops, such as in apple orchards, also showed that leaf sampling is less reliable for *B. rubrioculus* than the shoot-based sampling (Herbert et al. 1983; Rahmani et al. 2022). In contrast, leaf sampling is still the best method for detecting and sampling key webspinning mite, *Tetranychus pacificus* in almond orchards (Haviland et al. 2021).

Availability and use of efficient sampling are the foundation for developing an IPM program in cropping systems. Our study showed that shoot tuft sampling is the most effective method for monitoring *B. rubrioculus* in almond orchards during the growing season. In reference to our study results, we recommend a sampling strategy by selecting a minimum of ten trees representing the orchard border and interior, with five shoots from each tree, and jarred those shoots to letter-size (22 × 28 cm) white copy paper to dislodge the *B. rubrioculus* mites. The shoot tuft method should reasonably estimate the presence and density of *B. rubrioculus* in an almond orchard. It enables growers to make informed decisions, implement targeted control measures, and adopt sustainable practices for managing *B. rubrioculus* in almond orchards. Although this study provided growers and pest control consultants with a tool to monitor *B. rubrioculus*, any thresholds regarding the treatment decisions to address the in-season pest pressure need to be established in the future.

## Data Availability

The data can be found here, https://doi.org/10.5061/dryad.t76hdr8fg.
